# PPARα-Mediated Positive-Feedback Loop Contributes to Cold Exposure Memory

**DOI:** 10.1038/s41598-019-40633-3

**Published:** 2019-03-14

**Authors:** Soaad Alfaqaan, Tomoki Yoshida, Hiromi Imamura, Chihiro Tsukano, Yoshiji Takemoto, Akira Kakizuka

**Affiliations:** 1 Laboratory of Functional Biology, Graduate School of Biostudies, Kyoto University, Kyoto, 606-8501 Japan; 2 Department of Organic Chemistry, Kyoto University Graduate School of Pharmaceutical Sciences, Sakyo-ku Kyoto, Japan

## Abstract

Fluctuations in food availability and shifts in temperature are typical environmental changes experienced by animals. These environmental shifts sometimes portend more severe changes; e.g., chilly north winds precede the onset of winter. Such telltale signs may be indicators for animals to prepare for such a shift. Here we show that HEK293A cells, cultured under starvation conditions, can “memorize” a short exposure to cold temperature (15 °C), which was evidenced by their higher survival rate compared to cells continuously grown at 37 °C. We refer to this phenomenon as “cold adaptation”. The cold-exposed cells retained high ATP levels, and addition of etomoxir, a fatty acid oxidation inhibitor, abrogated the enhanced cell survival. In our standard protocol, cold adaptation required linoleic acid (LA) supplementation along with the activity of Δ-6-desaturase (D6D), a key enzyme in LA metabolism. Moreover, supplementation with the LA metabolite arachidonic acid (AA), which is a high-affinity agonist of peroxisome proliferator-activated receptor-alpha (PPARα), was able to underpin the cold adaptation, even in the presence of a D6D inhibitor. Cold exposure with added LA or AA prompted a surge in PPARα levels, followed by the induction of D6D expression; addition of a PPARα antagonist or a D6D inhibitor abrogated both their expression, and reduced cell survival to control levels. We also found that the brief cold exposure transiently prevents PPARα degradation by inhibiting the ubiquitin proteasome system, and starvation contributes to the enhancement of PPARα activity by inhibiting mTORC1. Our results reveal an innate adaptive positive-feedback mechanism with a PPARα-D6D-AA axis that is triggered by a brief cold exposure in cells. “Cold adaptation” could have evolved to increase strength and resilience against imminent extreme cold temperatures.

## Introduction

Environmental stimuli such as cold exposure or chronic dietary changes influence cellular responses, for instance, altered energy balance, gene expression, and composition or fluidity of lipid membranes^1–6^. It has previously been shown in various organisms that exposure to cold stimulates an increase in fat utilization^7,8^ and causes alterations in membrane fluidity through incorporation of unsaturated fatty acids into lipid membranes^9,10^. In addition, cold exposure also causes activation of the desaturase system^11^. A delta desturase, D6D, is a membrane-bound enzyme that catalyzes the synthesis of polyunsaturated fatty acids^12^ and is rapidly activated upon cooling^11^, possibly to increase survivability^13^. Desaturation of membrane lipids to maintain cellular integrity in cold temperatures might be common in both plants and mammals^14,15^.

Energy availability is highly influential to cellular responses and may shift energy metabolism, and trigger alterations in gene expression. PPARα is a known master regulator of lipid metabolism and is responsible for stimulating increases in fat utilization through peroxisomal and mitochondrial β-oxidation^16^. PPARα may be implicated in metabolic disease models such as metabolic syndrome, dyslipidemia and diabetes^17–19^.

The concept of cooling as a therapeutic tool can be found both in nature and in the medical field. Hibernation is an example where metabolic shifts and cellular responses are altered to maintain survivability. Considerable attention has been paid to the benefits of Therapeutic Hypothermia (TH) as a noninvasive therapy with the purpose of preserving the function of systems at risk of damage by decreasing temperatures to 32–34 °C for several days^20^. Previous studies have established that application of this treatment improved health outcomes in a multitude of medical conditions^21–25^. TH has also been observed to preserve and maintain glucose levels through alterations in metabolism^26,27^, and delays pro-inflammatory cytokine production^28^.

Notwithstanding the standard TH therapy duration and temperature, the outcomes of an acute and drastic drop in temperature have not been thoroughly investigated as a potential influencer of energy levels. Greater understanding of the molecular mechanisms that underlie the response to cooling at the cellular level will therefore assist these applications.

Herein, we explore the ability of a brief and drastic shift in temperature to enhance cellular viability and describe a new method of cellular cooling using a water bath system, by which cells are cooled from 37 °C to 15 °C in approximately 2 min. We uncover novel relationships among the short cold exposure, maintenance of intracellular ATP levels, mitochondrial membrane potential (MPP), and increased expression of PPARα and D6D. Collectively, these lead to enhanced cellular survivability.

## Results

**Figure 1:**
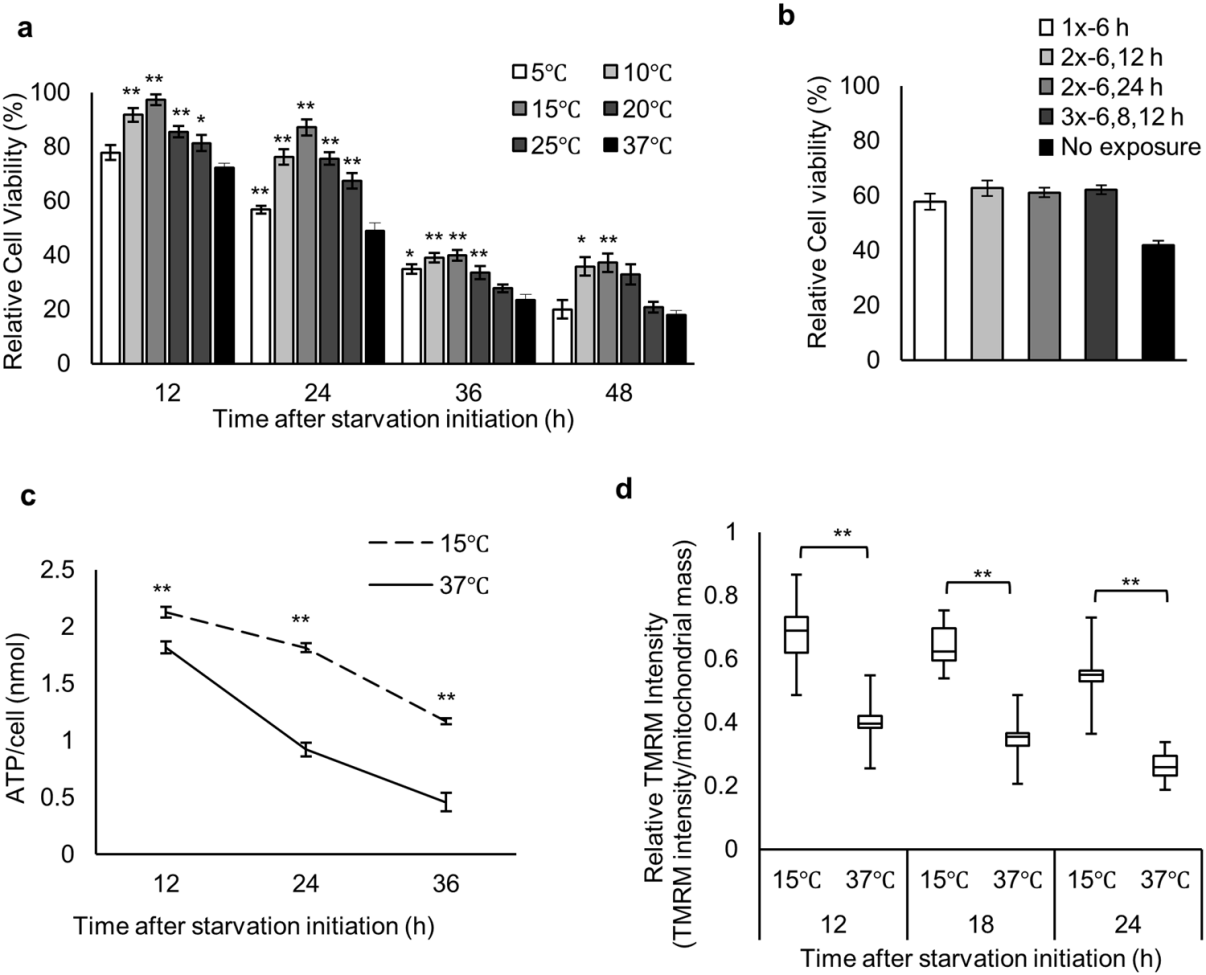
Cold exposure increases cell viability and maintains intracellular ATP levels and mitochondrial membrane potential following starvation. (**a**) Relative cell viability of (37 °C) and (5–25 °C) exposed cells at 6 h after initiation of starvation (*n* = 3). (**b**) Relative cell viability of cells treated with a single cold exposure (15 °C) at 6 h (1x-6 h), double exposures at 6 and 12 h (2x-6, 12 h), 6 and 24 h (2x-6, 24 h), and triple exposures at 6, 8, and 12 h (3x-6, 8, 12 h) after initiation of starvation, or no exposure (37 °C) (*n* = 3). **(c)** Intracellular ATP levels measured by luciferase assays (*n* = 3). (**d**) MMP in (37 °C) and (15 °C) cells. Mean values for cells grown continuously in DMEM at 37 °C were set at 1. Distribution of data based on the five-number summary: minimum, first quartile, median, third quartile, and maximum (12 h: 15 °C, *n* = 20 cells; 37 °C, *n* = 13. 18 h: 15 °C, *n* = 42; 37 °C, *n* = 42. 24 h: 15 °C, *n* = 40; 37 °C, *n* = 32). Assays for b were carried out at 30 h after initiation of starvation. Error bars represent SD. *P < 0.05, **P < 0.01 by unpaired two-tailed Student’s t-test.

We have analyzed the effects of starvation on ATP levels and cell death in cultured cells. After 3–4 days of incubation at 37 °C under starvation conditions, cell death occurred as a result of ATP depletion. However, in certain dishes significant numbers of cells were alive, even cultured with the same media (Supplementary Fig. 1a). After careful evaluation, we noticed that the dishes with live cells had been examined by microscopy at least once during the incubation. We hypothesized that cells can react to a brief exposure to cold temperature by creating a state of starvation resistance. We tested various conditions and identified a treatment consisting of an exposure to 15 °C for approximately 2 min, performed 6 h after the initiation of starvation, to be most effective at producing significant improvements in cell survival (Fig. 1a, Supplementary Fig. 1b). The influence of pH fluctuations during the treatment was negligible (Supplementary Fig. 1c,d). Multiple exposures of 2–3 × (15 °C for 2 min) at various time-points showed no significant increases in survival rate (Fig. 1b), indicating that the optimized condition, with a single 2-min exposure to 15 °C is enough to maximally protect cells against starvation.

**Figure 2:**
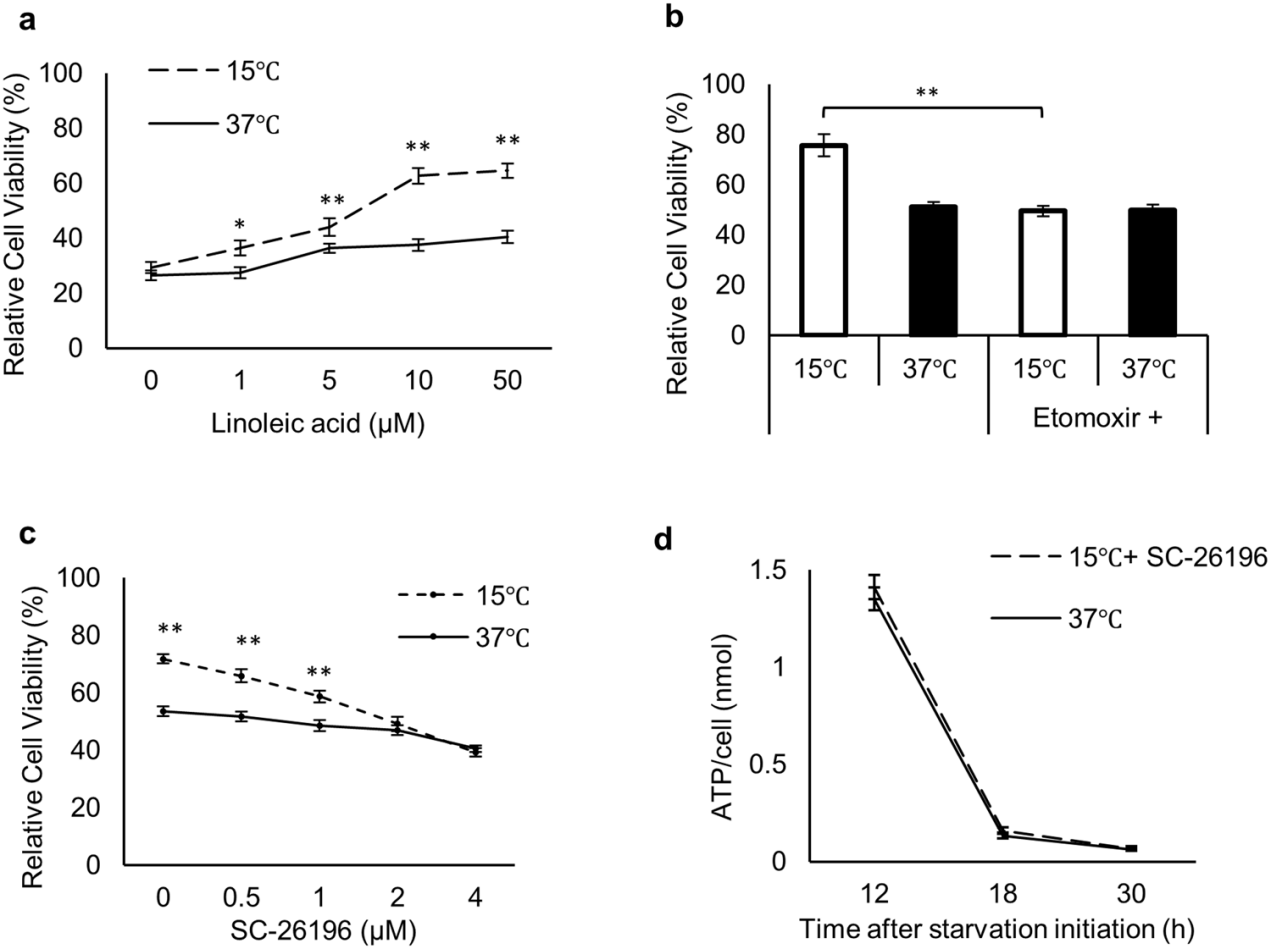
Linoleic acid supplementation along with D6D activity is required for enhanced cell viability and intracellular ATP maintenance (**a**) Dose response of LA supplementation on cell viability of non-exposed (37 °C) and cold-exposed (15 °C) cells (*n* = 3). (**b**) Viability of (15 °C) or (37 °C) cells treated with or without 100 μM etomoxir. (**c**) Dose response of SC-26196 on cell viability of (37 °C) and (15 °C) cells (*n* = 3). (**d**) Intracellular ATP levels measured by luciferase assays of (37 °C) and (15 °C) cells treated with 2 μM SC-26196 (15 °C + SC-26196) (*n* = 3). Assays for (**a**–**c**) were carried out at 30 h after initiation of starvation. Error bars represent SD. *P < 0.05, **P < 0.01 by unpaired two-tailed Student’s t-test.

Next, we measured intracellular ATP levels after the cold exposure and observed higher ATP levels in cold-exposed cells than in non-exposed cells during starvation (Fig. 1c, Supplementary Fig. 2a,b). ATP levels dropped by 40% in cold-exposed cells between 12–36 h, in contrast to a 75% drop in non-exposed cells (Fig. 1c). Mitochondria are crucial for ATP production, and changes in the MMP are reflective of cell health^29^. Thus, we next examined MMP using TMRM staining (Supplementary Fig. 2e)^30^. Consistent with the ATP levels, MMP levels in cold-exposed cells showed only a 14% decline at 18 h after cold exposure, whereas non-exposed cells experienced a 35% decline over the same time period (Fig. 1d, Supplementary Fig. 2e). Our starvation media (SM) contains only linoleic acid (LA) as a nutrient, and removal of LA abrogated the enhanced cell survival of cold-exposed cells (Fig. 2a). Treatment with etomoxir, a mitochondrial β-oxidation inhibitor, decreased the enhanced cell survival of cold-exposed cells to levels of non-exposed cells (Fig. 2b).

In addition to its potential as an energy source, LA metabolism yields several metabolites that serve important biological roles^31^. In this process, D6D is the rate-limiting enzyme responsible for metabolizing LA^32^. Thus, we investigated whether LA metabolites are involved in the cold adaptation. To this end, we applied SC-26196, a specific inhibitor of D6D^33^. Addition of the inhibitor to cold-exposed cells ablated the enhanced cell survival in a dose-dependent manner (Fig. 2c), as well as abrogated the effects of cold exposure for maintaining ATP levels (Fig. 2d, Supplementary Fig. 2c,d).

**Figure 3:**
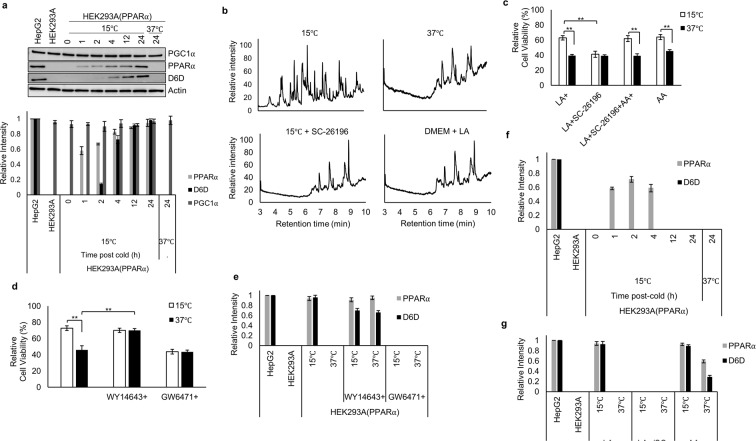
PPARα and D6D activities are essential for cold-induced survival. (**a**) PGC1α, PPARα, and D6D expression at various time points in (37 °C) and (15 °C) cells (*n* = 3). Blots for PGC1α and Actin were derived from the same gel, and blots for PPARα and D6D were derived from individual gels. Blots for individual proteins were generated using the same samples. (**b**) FAME analyses by GC-MS of (15 °C), (37 °C), (15 °C + 2 μM SC-26196), and cells in high glucose DMEM supplemented with 10 μM LA (*n* = 3). (**c**) Influence of FA supplementation on cell viability of (37 °C) and (15 °C) cells supplemented with 10 μM LA; 10 μM LA + 2 μM SC-26196; 10 μM LA + 10 μM AA + 2 μM SC-26196; and 10 μM AA (*n* = 3). (**d**,**e**) Effects of PPARα agonist (10 μM WY14643) or antagonist (1 μM GW6471) on cell viability (**d**) and PPARα and D6D expression levels (**e**) in (37 °C) and (15 °C) cells (*n* = 3). (**f**) PPARα and D6D expression at various time points without LA supplementation in (37 °C) and (15 °C) cells (*n* = 3). (**g**) Influence of FA supplementation on PPARα and D6D expression in (37 °C) and (15 °C) cells supplemented with 10 μM LA; 10 μM LA + 2 μM SC-26196; 10 μM AA (*n* = 3). Error bars represent SD. *P < 0.05, **P < 0.01 by unpaired two-tailed Student’s t-test. For gel source data, see Supplementary Fig. 4.

Surprisingly, expression of D6D was not detected in cells cultured in normal medium. We therefore investigated the regulation of its expression in response to cold exposure under LA supplementation. Western blot analysis revealed the induction of D6D expression in cold-exposed cells, with substantial expression observed at 4 h, and continued expression up to 24 h post-cold. In sharp contrast, D6D was not detected in non-exposed cells (Fig. 3a). To confirm D6D activity, we performed Fatty Acid Methyl Ester (FAME) analysis of lipid components by GC-MS of cold-exposed and non-exposed samples to compare the profiles of the chromatograms. FAME analysis revealed a large disparity between cold-exposed and non-exposed cells (Fig. 3b). Inhibition of D6D by SC-26196 in cold-exposed cells altered the chromatogram, rendering it like that of non-exposed cells (Fig. 3b). These results further support the notion that cold exposure induces D6D activity, thereby favoring LA metabolism.

Arachidonic acid (AA) is an LA metabolite with various biological functions^34–37^. We next investigated whether AA is involved in cold adaptation. Supplementation with AA recapitulated the cold-induced survival, even when D6D activity was inhibited (Fig. 3c), implying that the major role of D6D in cold adaptation is to produce AA. Among the known functions of AA^38,39^, we speculated that in cold adaptation it acts as an agonistic ligand for PPARα. PPARα is a master regulator of fatty acid (FA) metabolism^17,40^, and it induces genes specific for lipid metabolism, including D6D^41–43^. Somewhat surprisingly, PPARα was not detected in normal culture and starvation conditions at 37 °C. However, we observed PPARα expression from 1 h after the cold exposure, with subsequent D6D expression (Fig. 3a). By contrast, we observed steady-state, constitutive expression of peroxisome proliferator-activated receptor gamma coactivator 1-alpha (PGC1α), a coactivator or a protein ligand of PPARα^44,45^, in both cold-exposed and non-exposed cells (Fig. 3a). These results suggest that cold exposure primarily raises the PPARα protein level, promoting its activation through binding to PGC1α^19^. Activated PPARα in turn would induce D6D expression, leading to the production of AA, further activating PPARα. Consistent with this notion, application of a PPARα antagonist (GW6471) eliminated the expression of D6D along with the cold-induced survival (Fig. 3d,e, Supplementary Fig. 3a). Treatment with a PPARα agonist (WY14643) induced both PPARα and D6D expression, even without cold exposure (Fig. 3e, Supplementary Fig. 3a). In the presence of LA, we observed continuously increasing PPARα expression along with subsequent D6D induction after the cold exposure (Fig. 3a), however, this pattern was not observed in the absence of LA (Fig. 3f, Supplementary Fig. 3b). More interestingly, inhibition of D6D activity eliminated both D6D and PPARα expressions in cold-exposed cells (Fig. 3g, Supplementary Fig. 3c). Together, these results point to a positive-feedback mechanism between PPARα and D6D. It is interesting to note that HepG2 cells, derived from a human hepatocellular carcinoma, showed constitutive expression of both PPARα and D6D (Fig. 3a), which is not unexpected in cells that retain many liver-specific characteristics, including fat metabolism.

**Figure 4:**
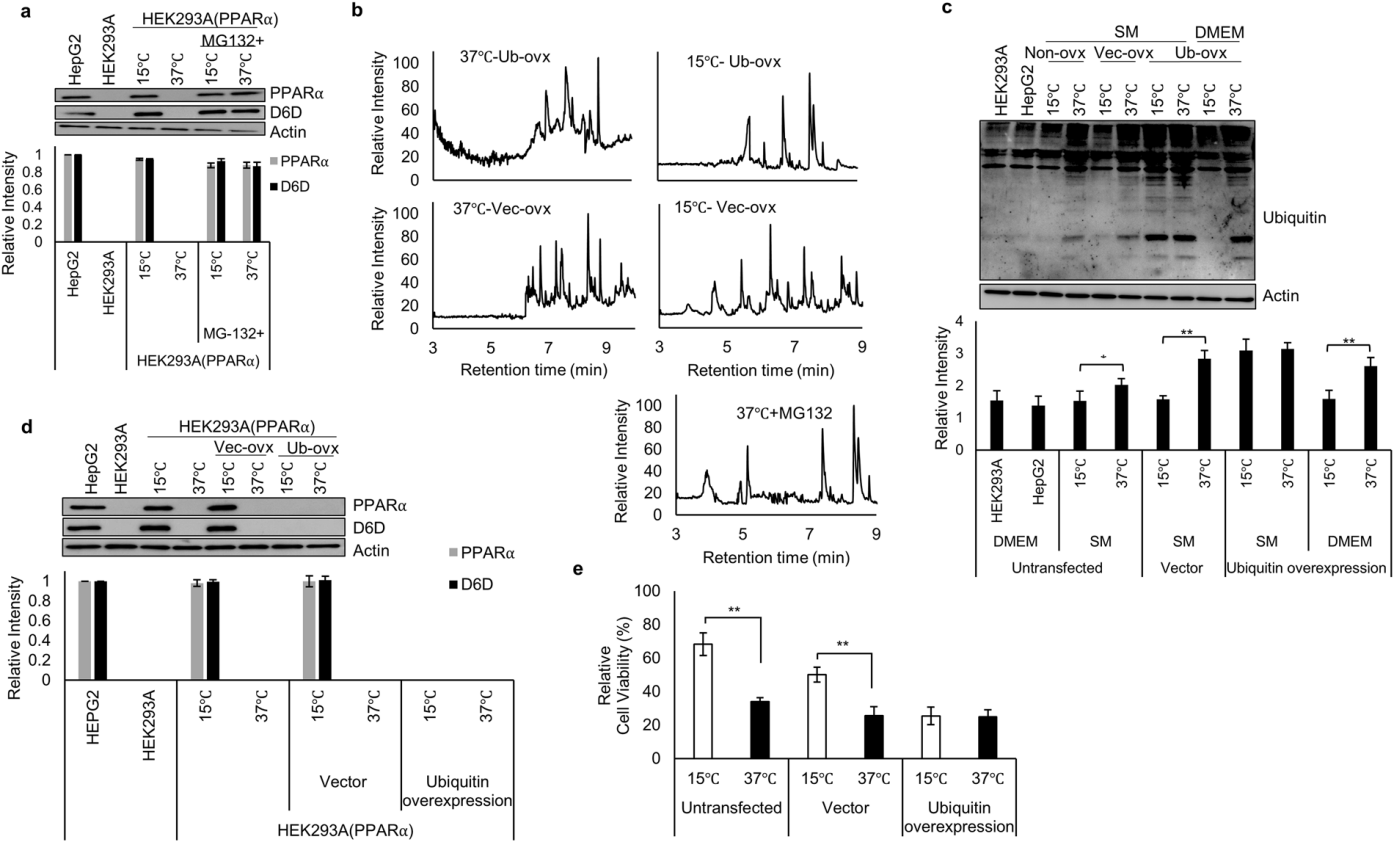
Cold exposure induces PPARα expression through inhibition of the UPS. (**a**) PPARα and D6D expression under treatment with 20 μM MG132. MG132 was added 6 h after initiation of starvation. (*n* = 3). (**b**) FAME analyses of (15 °C) or (37 °C) cells overexpressing ubiquitin. To determine the UPS involvement, cells were treated with 20 μM MG132 for 12 h before collection (*n* = 3). (**c**) Ubiquitination of proteins in untransfected cells (Non-ovx), cells overexpressing vector (Vec-ovx), or overexpressing ubiquitin (Ub-ovx). All cells were treated with 20 μM MG132 at 12 h before collection (*n = *3). (**d**) PPARα and D6D expression in untransfected cells, cells overexpressing vector (Vec-ovx), and cells overexpressing ubiquitin (Ub-ovx) (*n* = 3). (**e**) Influence of ubiquitin overexpression on cell viability in (15 °C) or (37 °C) cells (*n* = 3). Assays for (**c**–**e**) were carried out at 18 h after initiation of starvation. Blots for PPARα, D6D, Actin, and ubiquitin were derived from individual gels, using aliquots of the same samples. Error bars represent SD. *P < 0.05, **P < 0.01 by unpaired two-tailed Student’s t-test. For gel source data, see Supplementary Fig. 4.

PPARα is readily degraded by the ubiquitin proteasome system (UPS)^46,47^. Therefore, we examined PPARα levels in cells treated with MG132, a proteasome inhibitor, and observed sustained PPARα expression in both cold-exposed and non-exposed cells (Fig. 4a), confirming that constitutively translated PPARα is continuously degraded by the UPS. In addition to PPARα expression, D6D expression was also evident in cells treated with MG132 (Fig. 4a), along with downstream FA metabolites, reflecting the presence of D6D activity even in MG132-treated cells maintained at 37 °C (Fig. 4b). Thus, inhibition of PPARα degradation in non-exposed cells replicates the cold adaptation profile.

We then speculated that cold exposure interferes with the UPS. Indeed, we found a dramatic decrease in the accumulation of ubiquitinated proteins in cold-exposed cells compared to the non-exposed cells (Fig. 4c). Conversely, overexpression of ubiquitin increased the accumulation of ubiquitinated proteins, even in cold-exposed cells (Fig. 4c), eliminated PPARα and D6D expression in cold-exposed cells (Fig. 4d), and abrogated the cold-induced survival (Fig. 4e).

Consistently, cold-exposed cells overexpressing ubiquitin displayed no increase in FA metabolites (Fig. 4b). These results clearly indicate that cold exposure inhibits UPS activity, preventing PPARα degradation. Note that a decrease in ubiquitinated proteins was also observed in cells cultured in DMEM and exposed to cold (Fig. 4c), demonstrating that the ability of a cold exposure to inhibit the UPS is not dependent on a specific nutrient profile.

**Figure 5:**
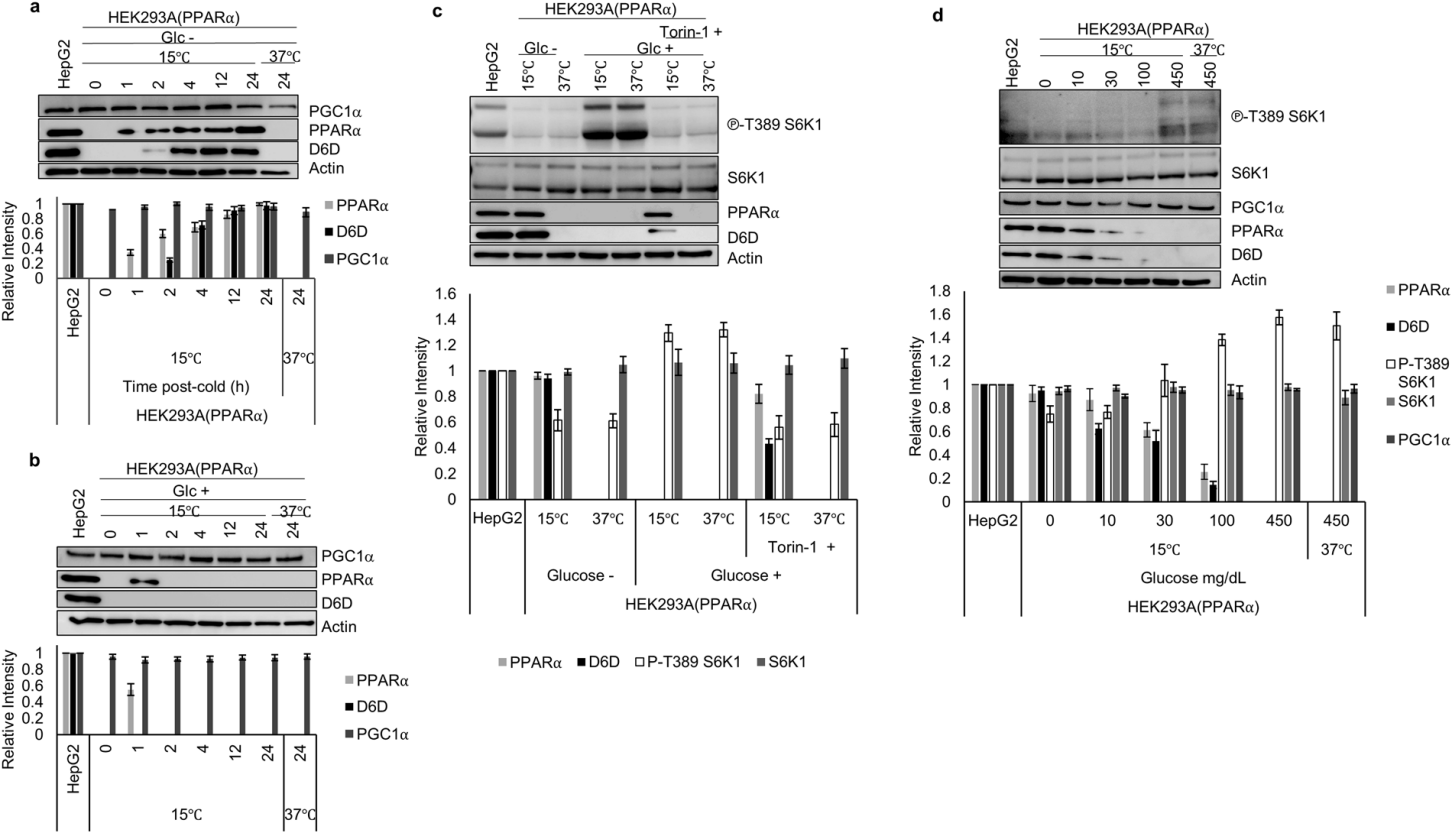
PPARα and D6D are not detectable under high glucose conditions. (**a**,**b**) Expression levels of PGC1α, PPARα, and D6D at various time points in (37 °C) and (15 °C) cells in DMEM without (**a**) and with (**b**) glucose (*n* = 3). (**c**) PPARα and D6D expression in (37 °C) and (15 °C) cells treated in DMEM ±glucose and ±1 μM Torin-1 (mTORC1 inhibitor). Torin-1 was added 24 h prior to collection, and cold exposed cells were treated at 18 h prior to collection (*n* = 3). Phospho-p70 S6 Kinase (Thr-389) represents phosphorylated S6 kinase at threonine 389 indicating mTORC1 activity, and p70 S6 kinase demonstrates endogenous levels of S6 kinase. (**d**) PPARα and D6D expression under varying doses of glucose (0–450 mg/dL) in (15 °C) or (37 °C) cells. Assays were carried out at 24 h after glucose deprivation (*n* = 3). (**a,b**) Blots for PGC1α and Actin were derived from the same gel, and PPARα and D6D were derived from individual gels, using aliquots of the same samples. (**c,d**) Blots were derived from individual gels, using aliquots of the same samples. Error bars represent SD. For gel source data, see Supplementary Fig. 4.

Cold exposure-induced PPARα and D6D expression was observed under DMEM glucose (−)/LA (+), but not in DMEM glucose (+)/LA (+), glucose (+)/LA (−), or glucose (−)/LA (−) at 24 h post-cold (Supplementary Fig. 4d). Closer investigation revealed that PPARα and subsequent D6D expression in DMEM glucose (−)/LA (+) were time-dependent (Fig. 5a). Surprisingly, a transient PPARα expression was detected even in DMEM glucose (+)/LA (+) at only 1 h post-cold (Fig. 5b). D6D, however, was not detected in this condition, implying that continuous expression as well as activation of PPARα does not occur under glucose (+) conditions.

A previous study revealed that the inhibition of the mammalian target of rapamycin 1 (mTORC1) is required for the activation of PPARα^48^. The activation of mTORC1 is influenced by nutrients such as glucose^49,50^, and triggers cellular responses by phosphorylating p70 S6 kinase^51^ at threonine 389, indicated as (phospho-p70 S6 Kinase) (Fig. 5c,d). Consistent with this data, addition of Torin-1, an mTORC1 inhibitor, even in DMEM glucose (+) led to clear PPARα and D6D expression after cold exposure (Fig. 5c). Furthermore, glucose doses ranging from 0 to 450 mg/dL showed an inverse relationship between PPARα and S6K phosphorylation levels. Although PPARα and D6D expression was not observed under the high glucose condition (450 mg/dL), both expressions were observed at relatively low glucose concentrations, up to 100 mg/dL glucose, after cold exposure (Fig. 5d).

## Discussion

Herein, we have uncovered an intricate regulatory relationship between an acute cold exposure and an increase in PPARα gene expression mediated through a PPARα-D6D-AA positive-feedback loop. During this study cold exposure appears to be initially sensed by the UPS and results in its inhibition, which leads to PPARα stabilization^46^. PPARα then transcriptionally activates genes involved in lipid metabolism, including D6D to produce AA, which further activates PPARα as its high affinity ligand. This gives rise to a positive-feedback loop between PPARα, D6D, and AA. This feedback loop continuously activates lipid metabolism, which is demonstrated by the maintained MPP levels to produce ATP. We refer to this phenomenon as “cold adaptation.” Furthermore, our data suggest that starvation further enhances this positive-feedback loop, as the presence of high amounts of glucose attenuates PPARα and D6D expression, and inhibition of mTORC1 was able to reverse this.

Cold exposure results in enhanced cellular dynamics^14^ and is associated with modulation of cell metabolism, gene expression, and cellular ATP levels^52–54^. Here we confirm these observations showing that, after cold exposure of cells to 15 °C for approximately 2 min, 6 h post starvation, cells do indeed modulate their metabolism and are capable of withstanding starvation under LA or AA supplementation. Single and multiple cold exposures lead to similar outcomes, indicating that this single exposure is fully capable of eliciting the cold exposure response in cells.

Adaptation is a fundamental survival strategy for organisms to cope with environmental changes and is most typically associated with neurons and the sympathetic nervous system. In this study we found that “cold adaptation” is a more fundamental type of cellular adaptation. HEK293A cells in culture acquire a quasi-stable cellular metabolic state that may be considered a form of cellular memory. In response to a brief exposure to cold in this case, this cellular “memory” sustained by a positive-feedback loop enhances cell viability and supports the notion of a fundamental type of cellular adaptation.

While the PPARα-D6D axis has been implicated under various environmental conditions, implication of the UPS by cold exposure leading to the enhancement of PPARα accumulation and activation has not previously been reported. The UPS regulates a multitude of systems. Hence, identifying and understanding the influencers of its activity can contribute to our general knowledge of the UPS itself and its controlling systems, such as cold adaptation. In addition, our data illustrates the presence of a PPARα-D6D-AA positive-feedback mechanism that is triggered by a brief cold exposure. This system may be utilized by mammals in cold seasons or hibernation, in which food is expected to be limited, and in which lipid metabolism is the primary source of fuel. It might be interesting to examine cells from such mammals to further investigate the mechanism driving resilience of cellular metabolic states.

## Materials and Methods

### Materials and Reagents

Materials, reagents, and vendors are as follows:

Dulbecco’s modified Eagle’s medium (DMEM), phenol red-free DMEM, and phosphate buffer saline (−) (PBS) (Nacalai Tesque, Kyoto, Japan); Earle’s balanced salt solution without glucose (EBSS) (prepared in our lab according to the recipe from Nacalai Tesque); fetal bovine serum (FBS) (Sigma, St. Louis, MO, USA); linoleic acid sodium salt (Nacalai Tesque); arachidonic acid sodium salt (Nacalai Tesque); penicillin/streptomycin mixed solution (Nacalai Tesque); fatty acid free bovine serum albumin (Nacalai Tesque); cell count reagent SF (Nacalai Tesque); Glo-Lysis buffer (Promega Corporation, Madison, WI, USA); luciferase-based ATP assay kit (Toyo B-Net, Tokyo, Japan); 35 mm glass-bottom dishes (Mattek, Ashland, MA, USA); MitoTracker Green FM and Tetramethylrhodamine, ethyl ester (TMRE) (Invitrogen, Carlsbad, CA, USA); Protein Assay Bicinchoninate Kit (Nacalai Tesque); HEPES (Dojindo, Japan); WY14647, GW6471, MG132, and etomoxir sodium salt (Cayman Chemical, USA); Fatty acid methyl ester mix (Sigma); Fatty acid methyl ester purification kit (06483) and fatty acid ester methylation kit (06482) (Nacalai Tesque); Torin-1 (Funakoshi, Japan); SuperSep Ace 5–20% (Wako, Osaka, Japan); Collagen Type IC (Cellmatrix, WAKO, Japan); SDS, 0.5% sodium deoxycholate (Nacalai Tesque) supplemented with 1 mM sodium disphosphate decahydrate, 1 mM NaF, 0.5 mM PMSF, 1 mM NaVO_4_, 1 × protease inhibitor cocktail (Nacalai Tesque), 10 mM β-glycerophosphate (Sigma), and 0.1% CHAPS (Dojindo, Kumamoto, Japan); a Protein Assay Bicinchoninate Kit (Nacalai Tesque); Lipofectamine 2000 (Life Technologies, Grand Island, NY); polyvinylidene difluoride (PVDF) membranes (Millipore, Billerica, MA, USA). All chemicals used were of analytical grade and were used as received without any further purification. All solutions were prepared with deionized water.

### Antibodies

Antibodies to phospho-p70 S6 kinase (Thr389) (9234), and p70 S6 kinase (9202) were purchased from Cell Signaling Technology (Danvers, MA, USA); the antibody to PGC1α (NBP1-0467622) was purchased from NovusBio (Littleton CO, USA); the antibody to D6D (ab170665) was purchased from Abcam; the antibody against PPARα (sc-9000) was purchased from Santa Cruz Biotech; antibodies against actin (MAB1501), and against ubiquitin (MAB1510) were purchased from EMD Millipore (Temecula, CA, USA). HRP-labeled secondary antibodies (GE Healthcare UK Ltd., Buckinghamshire, England) were used for visualization by enhanced chemiluminescence.

### Cells and Cell Culture

We established HEK293A cells continuously expressing ATeam-1.03^55,56^ (HEK293A-ATeam-1.03 cells). HEK293A and HEK293A-ATeam-1.03 cells were cultured and maintained in high glucose (450 mg/dL) DMEM, and HepG2 in low glucose (100 mg/dL) DMEM. Cell cultures were supplemented with 10% FBS and maintained at 37 °C and 5% CO_2_.

### Starvation and Cold Exposure

HEK293A cells were plated on 10 cm dishes (FPI, Japan) at a density of 10,000 cells/cm^2^ in high glucose DMEM, supplemented with 10% FBS. After 24 h, cells were gently washed twice with PBS, and then incubated in starvation medium (SM), composed of EBSS supplemented with 20 mM HEPES (pH 7.5), 10 μM linoleic acid sodium salt, 100 unit/mL penicillin, 100 μg/mL streptomycin, and 0.5% fatty acid free-BSA. After 6 h incubation period in SM, cells were removed from the incubator and directly placed on a cold-water bath. The temperature of the medium in the dish was monitored using a thermometer. After approximately 2 min on the water bath, the medium reached the desired temperature (15 °C) and the plate was immediately placed back into the incubator.

### Cell Viability Assays

Cell viability of HEK293A cells was determined using SF cell-counting reagent according to the manufacturer’s instructions (Nacalai Tesque). Measurements were carried out using a plate reader (ARVO multi-label counter, Perkin Elmer, Inc.). Cell viability was calculated as the ratio of treated cells divided by cells cultured in DMEM at 37 °C for 24 h. Cell viability was measured from independent trials.

### ATP Luciferase Assay

Intracellular ATP levels of HEK293A cells were determined according to the following protocol with slight modifications. Briefly, duplicate dishes were prepared for each individual sample, one for the ATP measurement, and one for cell count. For the ATP measurements, cells were lysed with Glo-lysis buffer for 5 min on ice after washing two times with pre-cooled PBS. ATP amount in the lysate was measured with a luciferase-based ATP assay kit (Toyo B-net). ATP measurements were obtained using a luminometer (ARVO multi-label counter), and the cell count was determined using a cell counter (countess II FL, Life technologies). ATP/cell values were calculated by dividing the total ATP amount in the lysate by the total cell number. ATP levels were measured from three independent trials.

### Imaging of Intracellular ATP Levels

HEK293A-ATeam-1.03 cells were plated at a density of 10,000 cells/cm^2^ on a 35 mm collagen-coated glass-bottom dish, in phenol red-free DMEM, and the starvation and cold exposure were performed as described above. Imaging was carried out as previously described^55^ using a Nikon Ti-E-PFS inverted microscope equipped with the following filter sets (Semrock, Rochester, NY) for dual emission ratio imaging, 438/24-DM458, 483/32 (CFP) or 542/27 (YFP) and a 40x objective (Nikon, Tokyo, Japan; CFI Plan Apo λ 40x: NA 0.95). Data were analyzed using MetaMorph analysis software (Molecular Devices, Sunnyvale, CA, USA). The YFP/CFP emission ratio was calculated by dividing YFP intensity by CFP intensity for each cell. ATP levels were measured from independent trials.

### Measurement of Mitochondrial Membrane Potential

Mitochondrial membrane potential (MMP) using HEK293A cells was measured according to the previous report^30^. HEK293A cells were plated on a 35 mm collagen-coated glass-bottom dish in phenol red-free DMEM, and the starvation and cold exposure were performed as described above. Cells were treated with 50 nM MitoTracker Green and 50 nM TMRM for 30 min at 37 °C. After washing out with pre-warmed phenol red-free starvation medium, imaging was carried out for measurement of florescent intensity of MitoTracker Green and TMRM from individual cells in phenol red-free starvation medium. MMP per cell was calculated by dividing the average intensities of TMRM by mitochondrial area as determined by signals from Mitotracker Green. Data were analyzed using MetaMorph analysis software. MMP was determined from cells of three independent trails.

### Western Blot Analysis

Cells were collected and lysed in RIPA buffer (150 mM NaCl, 1% NP40, 50 mM Tris-HCl (pH 7.6), 0.1% SDS, 0.5% sodium deoxycholate supplemented with 1 mM sodium disphosphate decahydrate, 1 mM NaF, 0.5 mM PMSF, 1 mM NaVO_4_, 1 × protease inhibitor cocktail 10 mM β-glycerophosphate, and 0.1% CHAPS. After sonication of the lysate on ice, the supernatant was collected by centrifugation at 4 °C at 15,000 rpm for 15 min. The protein content in the lysate was measured using a Protein Assay Bicinchoninate Kit (Nacalai Tesque), following the manufacturer’s instructions. Protein samples (35 μg) were prepared, loaded, and separated on SuperSep Ace 5–20% precast gels (Wako), and transferred onto polyvinylidene difluoride (PVDF) membranes (Millipore, Billerica, MA, USA). Western blot analysis detecting ubiquitin was carried out on a 15% gel.

For western blot analysis of PPARα expression, minimal expression of PPARα was detected in HEK293A cells, therefore, we overexpressed PPARα to fully visualize the capacity of cold exposure on enhancing PPARα expression (Supplementary Fig. 3e). Samples taken from three independent trails. For western blot analysis of PPARα, HEK293A(PPARα) were used, and control cells (HEK293A and HepG2) cultured in DMEM at 37 °C were harvested 24 h after seeding. Relative protein expression levels were normalized to actin. Values for HepG2 cells were set at 1. Unless specified otherwise, western blot samples were collected 30 h after initiation of starvation.

Samples in Figs 3a,c,d,e, 4a,c,d, and 5 were all supplemented with 10 µM LA, and samples in Fig. 3f were not supplemented with LA.

### Fatty Acid Methyl Ester (FAME) Composition Analysis

GC analysis using electron capture detection was used to characterize the FA composition of cell samples. FA extraction was carried out according to this report^57^. Briefly, 18 h after the initiation of starvations, HEK293A cells were pelleted by centrifugation at 7,500 rpm for 5 min at 4 °C. Lipids were then extracted by addition of 600 μL cold acetone followed by three rounds of vortexing for 1 min each, freezing with liquid nitrogen, and ultrasonication for 5 min. Cells were then incubated at −20 °C for 1 h, followed by centrifugation at 15,000 rpm at 4 °C for 15 min. The supernatant was collected into a fresh tube and placed on ice. The pellet is resuspended in 400 μL of methanol: water: formic acid (86.5:12.5:1.0), followed by vortexing for 1 min, and 10 min of ultrasonication in a water bath. The sample was placed at −20 °C for 1 h, followed by centrifugation at 15,000 rpm at 4 °C for 15 min. The supernatant was collected and combined with the supernatant from the first extraction. The sample was dried under nitrogen followed by methylation and purification using a FA methylation kit, and FA purification kit, respectively (Nacalai Tesque), according to manufacturer’s protocol. The sample was injected at a concentration of 1 mg/mL into a GC-MS system consisting of a GCMS-QP2010/Parvum2 (Shimadzu) attached to a DB-5 MS column (Agilent technologies). The flow rate of the Helium gas as a carrier was 1.25 mL/min. Samples taken from three independent trails.

### Transfection

HEK293A cells were cultured in 10 cm dishes in DMEM supplemented with 10% FBS and incubated at 37 °C for 24 h. The total amount of DNA in each transfection was adjusted to 7.0 μg/dish for pcDNA3.1 FLAG-ubiquitin, and 10.5 μg/dish for vector (pcDNA3.1(−)), and pLXSN PPARα-5974. Co-transfections were performed simultaneously with the DNA concentrations. Transfections were performed using Lipofectamine 2000 (Life Technologies, Grand Island, NY) according to the manufacturer’s recommendations.

## Supplementary information

Supplementary Information

## Data Availability

The datasets generated during and/or analyzed during the current study are available from the corresponding author on reasonable request.
