# Supplementary material for: PPARα-Mediated Positive-Feedback Loop Contributes to Cold Exposure Memory
Source: Sci Rep. 2019 Mar 14;9:4538. doi: 10.1038/s41598-019-40633-3 (PMC6418111; doi:10.1038/s41598-019-40633-3)
Supplement: Supplementary file 1 — Supplementary Information [file 41598_2019_40633_MOESM1_ESM.pdf]

PPARα-Mediated Positive-Feedback Loop Contributes to Cold Exposure Memory

Soaad Alfaqaan[1], Tomoki Yoshida[1], Hiromi Imamura[1], Chihiro Tsukano[2], Yoshiji Takemoto[2], Akira Kakizuka[1] *

[1] Laboratory of Functional Biology, Graduate School of Biostudies, Kyoto University, Kyoto 606-8501, Japan

[2] Department of Organic Chemistry, Kyoto University Graduate School of Pharmaceutical Sciences, Sakyo-ku, Kyoto, Japan

*Correspondence: kakizuka@lif.kyoto-u.ac.jp

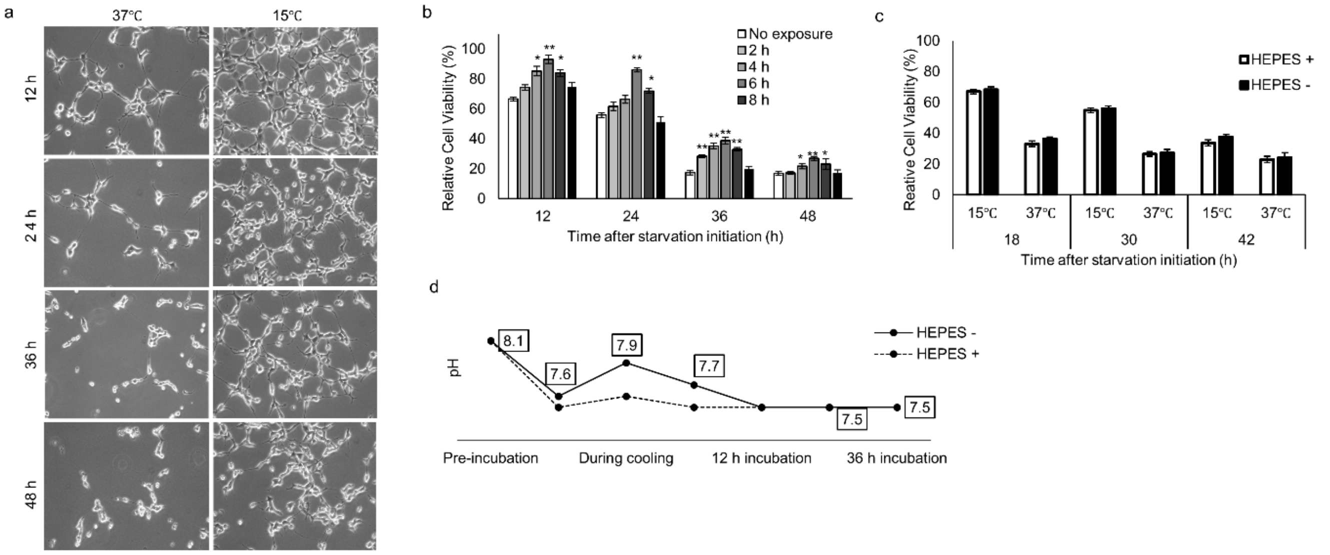

Supplementary Fig. 1. Cold exposure increases cell viability.
(**a**) Morphology of non-exposed (37°C) and cold-exposed (15°C) cells at various time points after initiation of starvation. Cold exposure was carried out for 2 min at 6 h after initiation of starvation. (**b**) Influence of time of treatment (2 min, 15°C) on cell viability. Cold exposure was carried out at 2, 4, 6, 8, or 12 h after initiation of starvation (n = 3). (**c**) Impact of pH on the effectiveness of cold exposure on cell viability. Cell viability of non-exposed (37°C) and cold-exposed (15°C) cells starved in medium supplemented with or without HEPES (n = 3). (**d**) Measurements of the pH of the medium at various time points during the experiment. Error bars represent SD. $*P <0.05$, $**P <0.01$ by unpaired two-tailed Student's t-test.

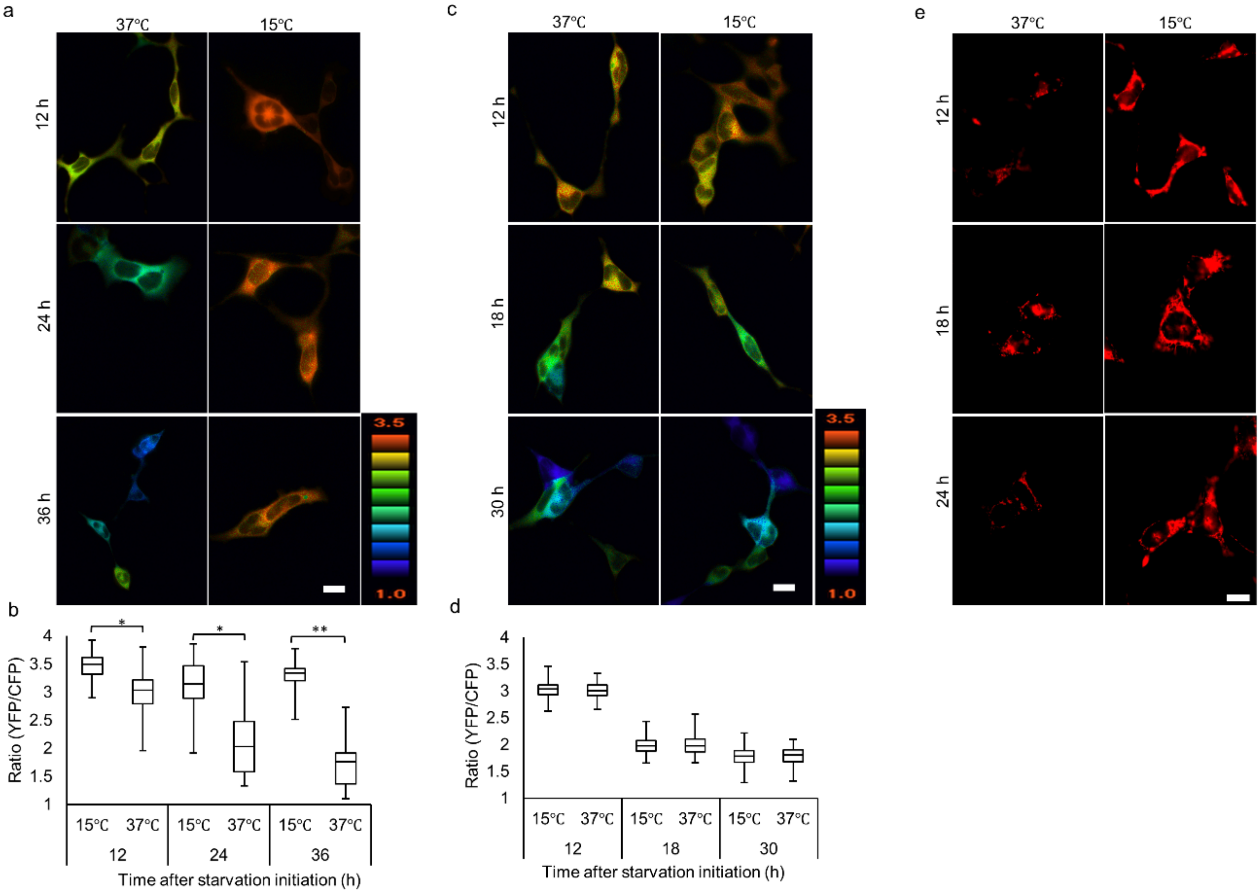

Supplementary Fig. 2. Cold exposure maintains intracellular ATP levels and mitochondrial membrane potential. (**a-d**) Measurement of ATP levels by ATeam1.03. (**a**) Visualization of intracellular ATP levels in non-exposed (37°C) and cold-exposed (15°C) cells during starvation. **(b)** Quantification of intracellular ATP levels (measured as the ratio of YFP/CFP). Distribution of data based on the five-number summary: minimum, first quartile, median, third quartile, and maximum (12 h: 15°C, n = 92; 37°C, n = 111; 24 h: 15°C, n = 107; 37°C, n = 101; 36 h: 15°C, n = 105; 37°C, n = 80). (**c**) Visualization of intracellular ATP levels in non-exposed (37°C) and cold-exposed (15°C) cells treated with 2 μM SC-26196. SC-26196 was added at 6 h after initiation of starvation. (**d**) Quantification of intracellular ATP levels (measured as the ratio of YFP/CFP). Distribution of data based on the five-number summary: minimum, first quartile, median, third quartile, and maximum from independent trials (12 h: 15°C, n = 113; 37°C, n = 70. 18 h: 15°C, n = 114; 37°C, n = 108. 30 h: 15°C, n = 114; 37°C, n = 105). (**e**)Visualization of MMP in non-exposed (37°C) and cold-exposed (15°C) cells at 12, 18, and 24 h after initiation of starvation. Scale bar represents 10 μm. *P <0.05, **P <0.01 by unpaired two-tailed Student's t-test. Color scale is representative of relative ATP levels. Scale bar represents 10 μm.

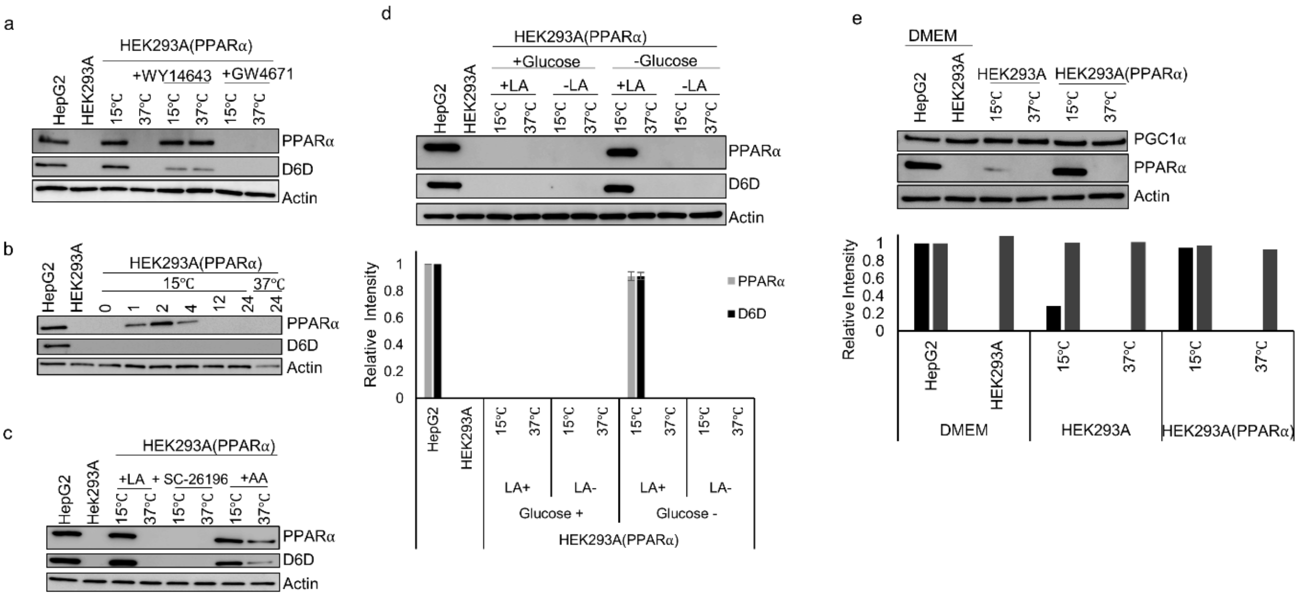

Supplementary Fig. 3. Expression levels of PPARα and D6D in different culture conditions. (**a**) Response to PPARα agonist (10 μM WY14643) or antagonist (1 μM GW6471) on PPARα and D6D expression levels in non-exposed (37°C) and cold-exposed (15°C) cells. (**b**) Time of induction of PPARα and D6D expression at various time points without LA supplementation during starvation in non-exposed (37°C) and cold-exposed (15°C) cells. (**c**) Influence of FA supplementation on PPARα and D6D expression in non-exposed (37°C) and cold-exposed (15°C) cells supplemented with 10 μM LA, 10 μM LA +2 μM SC-26196, or 10 μM AA. (**d**) PPARα and D6D expressions were limited to glucose (-)/LA (+) conditions. PPARα and D6D expression in non-exposed (37°C) and cold-exposed (15°C) cells treated in DMEM with or without glucose and/or LA supplementation. All cells were collected at 24 h post-treatment. (*n* =3). (**e**) Comparison of PPARα expression in HEK293A cells and HEK293A(PPARα) cells exposed to cold (15°C). All cells were collected at 24 h post-treatment (*n* = 3). All blots were derived from individual gels using aliquots of the same samples. Values for HepG2 cells were set at 1. Error bars represent SD. For gel source data, see Supplementary Fig. 4.

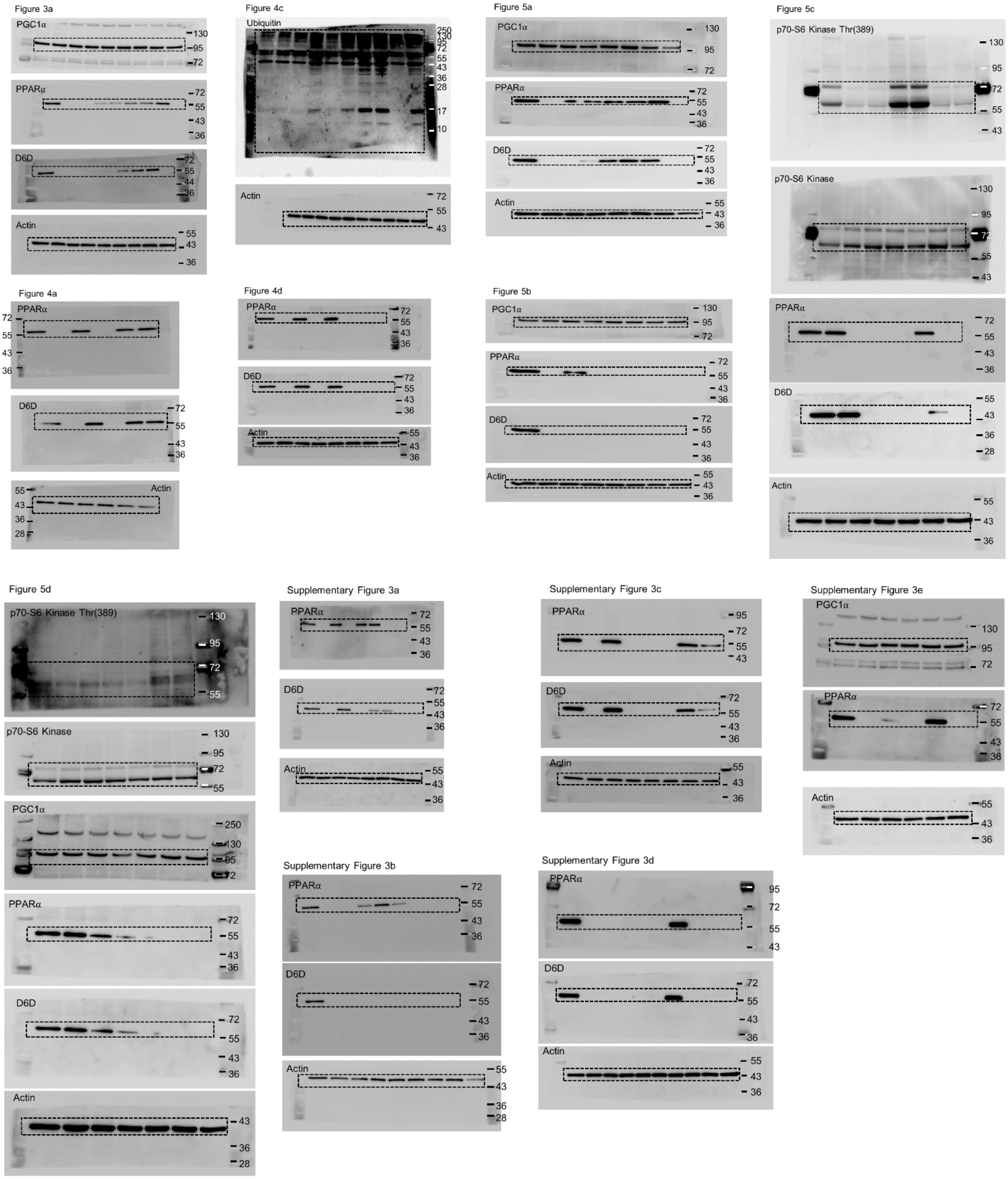

Supplementary Fig. 4. Unedited images of western blots.
